# Environmental stressors, sleep, and a visit from St. Nicholas

**DOI:** 10.1093/sleepadvances/zpad048

**Published:** 2023-11-28

**Authors:** Michael G Smith, Mathias Basner

**Affiliations:** School of Public Health and Community Medicine, Institute of Medicine, Sahlgrenska Academy, University of Gothenburg, Gothenburg, Sweden; Unit for Experimental Psychiatry, Division of Sleep and Chronobiology, Department of Psychiatry, University of Pennsylvania Perelman School of Medicine, Philadelphia, USA

The importance of adequate quantity and quality of sleep is increasingly recognized by clinicians, policy makers, and the public as a critical cornerstone of good physical and mental health. Although progress is being made, the crucial role of sleep disturbance by environmental stressors is often neglected in public health discourse, as was highlighted recently by Lim et al. [1] To further stimulate this discussion, we here highlight the major clinical and public health issues of adequate quantity and quality of sleep via a narrative review on the effects of environmental stressors on sleep, framed within the context of the 1823 poem A Visit From St. Nicholas by Clement Clarke Moore [2].

## Stanza 1

‘Twas the night before Christmas, when all through the house
Not a creature was stirring, not even a mouse;
The stockings were hung by the chimney with care,
In hopes that St. Nicholas soon would be there;

This calm and quiet environment is a key component of promoting good sleep. Unfortunately for the narrator, the recuperative benefits are soon to be substantially reduced by multiple environmental stressors. As described by the social-ecological model of sleep health, physical and mental health effects of sleep are influenced by individual-, social-, and societal-level factors [3]. Myriad environmental stressors exist across each of these domains, and the prevalence of many of such stressors is even greater now than when the poem was penned.

## Stanza 2

The children were nestled all snug in their beds;
While visions of sugar-plums danced in their heads;
And mamma in her ‘kerchief, and I in my cap,
Had just settled our brains for a long winter’s nap,

The most vivid dreaming, which may or may not involve sugar-plums, occurs during rapid eye movement (REM) sleep. Non-REM sleep is further subdivided into N1 sleep (transitional “light” sleep), N2 (“intermediate”) sleep, and N3 (“deep”) sleep, also known as slow wave sleep (SWS). SWS is important for synaptic homeostasis memory consolidation [4], insulin sensitivity [5], growth hormone secretion [6], glymphatic clearance [7], and possibly longevity generally [8]. The functional role of REM sleep is less well understood, but may include brain development, formation and consolidation of memory, synaptic pruning and brain plasticity, and stimulating the central nervous system in preparation for wakefulness [9]. Disturbance of SWS or REM sleep by environmental stressors can therefore interfere with processes crucial for health.

Warm bedclothes during winter can help to maintain an optimal thermal environment for sleep. Temperature has an integral role in regulating sleep, and temperatures that are too cold or too warm negatively impact on sleep quality [10]. The effects of heat on sleep may be exacerbated by high humidity, which could impair thermoregulation by evaporative cooling [11]. These findings have implications for the effects of climate change, and warming temperatures are projected to reduce the total amount of sleep globally and increase the risk of insufficient sleep [12].

## Stanza 3

When out on the lawn there arose such a clatter,
I sprang from my bed to see what was the matter.
Away to the window I flew like a flash,
Tore open the shutters and threw up the sash.

Here, we observe disturbance of sleep by outdoor noise. Environmental noise from road, aircraft, and railway traffic is highly prevalent in urbanized society. Consequently, an estimated 8 million European adults suffer from sleep disturbance due to traffic noise [13]. In 2011, the WHO estimated that disturbed sleep by noise led to an annual loss of 903 000 healthy life years in Europe [14]. The actual burden is likely higher, due to continued increases in noise levels and reliance of previous estimates on self-reported data.

## Stanza 4

The moon on the breast of the new-fallen snow,
Gave a lustre of midday to objects below,
When what to my wondering eyes did appear,
But a miniature sleigh and eight tiny reindeer,

Light is another key environmental stressor for sleep. In this context, we refer to artificial light at night, rather than natural daylight which has an important role in keeping circadian rhythms entrained [15]. Photoreceptors in the retina transmit light information to retinal ganglion cells. Melanopsin in these cells drives suppression of the sleep-promoting hormone melatonin from the pineal gland [16]. Thus, light exposure can delay secretion of melatonin, leading potentially to adverse effects on sleep and circadian rhythms [17, 18]. Further, light exposure during sleep can impair cardiometabolic function [19]. A recent expert consensus statement recommended that in order to maintain appropriate circadian, neuroendocrine, and neurobehavioral function, light should not exceed 10 lux in the 3 h before bedtime, and should not exceed 1 lux (at the eye) in the bedroom during sleep [17].

## Stanza 5

With a little old driver so lively and quick,
I knew in a moment he must be St. Nick.
More rapid than eagles his coursers they came,
And he whistled, and shouted, and called them by name:

Saint Nicholas is the patron saint of, among others, children and students. Children are recognized as a vulnerable group to the effects of disturbed sleep, because of the importance of sleep for brain development [20]. Furthermore, children often sleep outside of the official nighttime hours when exposures such as noise may be regulated, and so have an increased exposure burden during their sleep period.

Despite sleep’s importance for physical and mental health and performance, it is often sacrificed in favor of familial, social, occupational, and/or educational obligations [21]. It is unsurprising that a large proportion of students do not obtain enough sleep [22], likely due to these social stressors. Sleep is crucial for learning because it helps maintain critical cognitive processes such as attention, working memory, creativity, and problem solving [23–26]. Learning may be further promoted by the encoding of short-term memory into long-term storage during sleep [27].

## Stanza 6

“Now, Dasher! now, Dancer! now Prancer and Vixen!
On, Comet! on, Cupid! on, Donner and Blitzen!
To the top of the porch! to the top of the wall!
Now dash away! dash away! dash away all!”

Traffic is not the only source of environmental noise; here, we see an example of neighborhood noise. In addition to the ruckus of noisy passers-by, other neighborhood noises include barking dogs, industry and commerce, parties, church bells, wind farms, compressors, generators, and heat pumps, to name but a few.

## Stanza 7

As leaves that before the wild hurricane fly,
When they meet with an obstacle, mount to the sky;
So up to the housetop the coursers they flew
With the sleigh full of toys, and St. Nicholas too—

Flying vehicles are a prevalent source of environmental noise. In epidemiological studies, aircraft noise is associated with higher levels of self-reported sleep disturbance than road or railway traffic of the same noise level [28]. On the other hand, acute physiological effects in response to noise, including event-related awakenings, cortical arousal, and elevations of heart rate, have been found in the laboratory to be lower for aircraft noise than other traffic modes [29]. This may be because of the more rapid noise onset of road and rail noise events, and also differences in the frequency spectrum of the noise, highlighting that response is affected by more than the noise level alone.

## Stanza 8

And then, in a twinkling, I heard on the roof
The prancing and pawing of each little hoof.
As I drew in my head, and was turning around,
Down the chimney St. Nicholas came with a bound.

Cognizance of the stressor and attitude toward its source can impact on the stressor’s potential to cause disturbance or annoyance. Hearing reindeer prancing about on one’s roof would conceivably raise concerns of damage to property, and thus induce a stronger psychological response than the noise level alone would suggest. For example, annoyance of people exposed to vibration from nearby railway lines is strongly mediated by concern of damage to property by the vibration [30].

Relationships between night-time noise exposure and self-reported sleep disturbance are not found when sleep disturbance questions are noise agnostic [28]. By identifying noise as an exposure of interest, questionnaire responses can be influenced by individual, non-acoustical factors arising from attitude toward the source mentioned. For example, recent field studies of noise from wind turbines found no direct relationship between noise levels and sleep, but did find associations between wind turbine noise annoyance and sleeping less deeply [31] or with reduced self-reported sleep efficiency [32].

## Stanza 9

He was dressed all in fur, from his head to his foot,
And his clothes were all tarnished with ashes and soot;
A bundle of toys he had flung on his back,
And he looked like a pedler just opening his pack.

Ashes and soot are indicators of fuel burning. Fossil fuels emit airborne pollutants including fine particulate matter (PM_2.5_), carbon dioxide (CO_2_), and nitrogen oxides (NO_*x*_). Compared to noise and light, there have been comparatively few studies examining the direct impact of air pollutants/air quality on sleep, especially studies considering indoor levels [33]. While associations between air pollution and poor sleep quality have been found [34], evidence is most compelling for increased risk of developing sleep-disordered breathing, including obstructive sleep apnea (OSA) [33], and for more severe apnea [35].

## Stanza 10

His eyes—how they twinkled! his dimples, how merry!
His cheeks were like roses, his nose like a cherry!
His droll little mouth was drawn up like a bow,
And the beard on his chin was as white as the snow;

Santa’s complexion may in part be a result of decades of quaffing glasses of brandy, sherry, and whisky left out by well-meaning parents. Alcohol is a depressant that can decrease sleep latency and may increase the amount of SWS in the first half of the night. However, it can also significantly increase sleep fragmentation and wakefulness during the second half of the night, and increase sleep-related respiratory disturbances [36].

White hair indicates Santa is likely an older individual. Sleep changes over the life course, both in duration and macrostructure. From early adulthood, sleep duration and sleep efficiency decrease, and the prevalence of insomnia symptoms increases [37]. Older individuals may therefore be at increased vulnerability to further disruption of sleep by environmental stressors, although this may be offset somewhat by lower sleep need.

## Stanza 11

The stump of a pipe he held tight in his teeth,
And the smoke, it encircled his head like a wreath;
He had a broad face and a little round belly
That shook when he laughed, like a bowl full of jelly.

Santa, being overweight and a smoker, is at increased risk for disturbed sleep. Smoking is a risk factor for sleep-disordered breathing [38], and smokers are more likely to suffer from insomnia-like sleep impairments [39, 40]. The relationship between sleep and BMI is likely bidirectional. Because of the modulating role of sleep for neuroendocrine and metabolic function and the regulation of leptin and ghrelin, insufficient sleep is associated with increased risk of obesity [41]. On the other hand, obesity increases the risk of disturbed sleep. Obesity, especially central obesity, is a major risk factor for the development of OSA [42, 43]. OSA is characterized by repeated reduction or cessation of airflow due to airway collapse, leading to hypoxemia, sympathetic nervous system activation, and sleep fragmentation. Mechanisms responsible for increased OSA risk due to obesity include narrowing of the upper airway (which is, therefore, more collapsible), changes in upper airway function, reduced lung volume, and changes in the balance between ventilatory drive and load [42].

## Stanza 12

He was chubby and plump, a right jolly old elf,
And I laughed when I saw him, in spite of myself;
A wink of his eye and a twist of his head
Soon gave me to know I had nothing to dread;

Despite that Santa is an elf, one can assume he nevertheless needs to sleep. In all animals in which sleep has been studied, sleep has been observed in one form or another [44]. This highlights that sleep is essential for life. One hypothesis proposes that sleep is the price we pay for brain plasticity, i.e. the ability to change its wiring in response to experiences made during the wake period [45].

Santa manages to assure the narrator they can relax. Psychosocial stress is a significant source of sleep disturbance, and is associated with shortened sleep and sleep fragmentation [46]. Curtailed sleep can lead to elevations in evening cortisol concentrations [47], likely exacerbating the problem.

## Stanza 13

He spoke not a word, but went straight to his work,
And filled all the stockings; then turned with a jerk,
And laying his finger aside of his nose,
And giving a nod, up the chimney he rose;

Shift workers are required to sleep at times of day when they are exposed to higher levels of environmental stressors, such as noise and light, placing them at higher risk of disturbance compared to sleeping at night. Further, night shift work is per se associated with poor sleep quality and circadian misalignment [48]. Meta-analyses have found that shift work is associated with increased risk for cardiovascular disease, cancers, type 2 diabetes, stroke, weight gain, and cognitive impairment [49–51]. However, these findings for chronic diseases are suggestive but not conclusive, due to high heterogeneity across individual studies. In the particular case of Santa, his extended hours and working at times when the circadian system is not promoting alertness places him at increased risk for crashing the sleigh toward the end of his shift, over North America [52].

Racial and ethnic minorities and populations with low socioeconomic status are more likely to be shift workers. As highlighted by Jackson et al. [53], deficiencies in sleep health among these disproportionately affected groups could play an important role in driving health disparities.

## Stanza 14

He sprang to his sleigh, to his team gave a whistle,
And away they all flew like the down of a thistle.
But I heard him exclaim, ere he drove out of sight—
“Happy Christmas to all, and to all a good night!”

A good night is one with an undisturbed and restful sleep period. At the individual level, this can be accomplished by adopting good sleep hygiene practices. Keeping the bedroom quiet, dark and cool, avoiding light and stimulants before bedtime, and maintaining a regular sleep schedule (i.e. going to bed and waking at the same time every evening and morning) all help to promote sleep. Improved sleep can benefit multiple aspects of health and well-being, not least mental health [54]. Increased recognition of the importance of good sleep hygiene practices, as well as mitigation of relevant stressors by authorities, can benefit patient outcomes, public health, and indeed our own physical and mental well-being. Happy Christmas to all, and to all a good night!
